# Infection and myelodysplasia: A case report of GATA2 deficiency in a South African patient

**DOI:** 10.1002/ccr3.7075

**Published:** 2023-03-14

**Authors:** Erica‐Mari Nell, Helena Cornellissen, Katherine Hodkinson, Michael F. Urban, Fatima Cassim Bassa, Fatima Bibi Fazel, Tracey Wiggill, Semira Irusen, Zivanai C. Chapanduka

**Affiliations:** ^1^ Division of Haematological Pathology, Faculty of Medicine and Health Sciences Stellenbosch University and National Health Laboratory Service, Tygerberg Hospital Cape Town South Africa; ^2^ Department of Molecular Medicine and Haematology, Faculty of Health Sciences University of the Witwatersrand and National Health Laboratory Service Johannesburg South Africa; ^3^ Division of Molecular Biology and Human Genetics, Faculty of Medicine and Health Science Stellenbosch University Stellenbosch South Africa; ^4^ Division of Clinical Haematology, Department of Internal Medicine, Faculty of Medicine and Health Sciences Stellenbosch University and Tygerberg Hospital Cape Town South Africa

**Keywords:** GATA2 deficiency, inborn errors of immunity, lymphoedema, myelodysplastic syndrome, myeloid neoplasms with germline predisposition, non‐tuberculous mycobacterium

## Abstract

Rare diseases often result in delays in diagnosis. It is important to recognize conditions that have features of both inborn errors of immunity and predispose to myeloid neoplasia. Here we report a patient with GATA2 deficiency that presented with disseminated non‐tuberculous mycobacterial infection and pancytopenia secondary to myelodysplastic syndrome.

## Graphical abstract

We present a patient with GATA2 deficiency who has features of an inborn error of immunity and myelodysplasia, highlighting the role of next‐generation sequencing in the diagnostic approach.

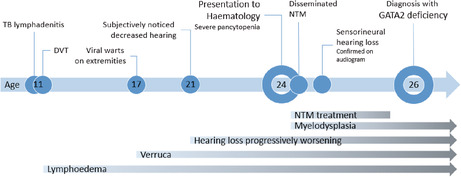

## 1 INTRODUCTION

Myeloid malignancies such as myelodysplastic syndrome (MDS) and acute myeloid leukemia (AML) typically occur as a sporadic disease due to acquired somatic mutations. However, with increased access to molecular tests, the role of inherited and de novo germline mutations as important drivers in myeloid malignancy are increasingly being recognized.

Identifying patients with germline variants that predispose them to MDS/AML requires recognition of out‐of‐the‐ordinary patterns of clinical features and organ dysfunction.^1^ Severe, persistent, unusual and/or recurrent mycobacterial infections as well as peripheral blood cytopenia, in the absence of secondary immunodeficiency, should raise concern for a possible inborn error of immunity (IEI).^2^ In addition, severe and/or persistent cytopenias in the absence of an identifiable cause is an important indication for bone marrow examination (BME).^1^, ^3^

In this case report we describe a patient with a history of disseminated non‐tuberculous mycobacterial infection (NTM), lymphoedema, persistent cutaneous viral warts and pancytopenia, who was diagnosed with germline GATA2 deficiency.

## 2 CASE REPORT

**FIGURE 1:**
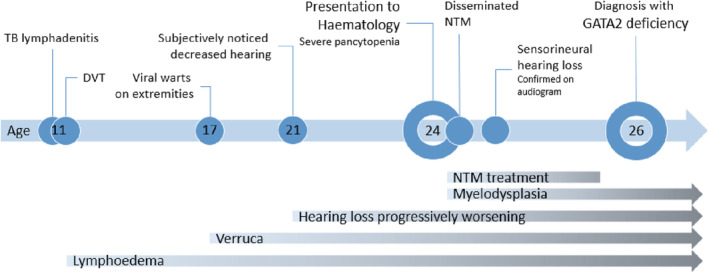
A timeline of symptoms and signs in a patient with GATA2 deficiency. DVT, deep vein thrombosis, NTM, non‐tuberculosis mycobacterial infection, TB, tuberculosis.

A 24‐year‐old, mixed race, HIV‐negative male was referred to Clinical Hematology at Tygerberg Hospital for investigation of a persistent severe pancytopenia. He had a history of tuberculosis (TB) lymphadenitis at the age of 11 for which he received the normal duration of 9 months of anti‐mycobacterial therapy. He also received anticoagulation for a concurrent deep vein thrombosis (DVT) of the left lower limb that resulted in swelling of the leg. The swelling persists to date, consistent with lymphoedema. He also gives a history of progressive sensorineural hearing loss from age 21 (Figure 1). He reported no current or previous respiratory symptoms.

He was born at 30 weeks' gestation and was hospitalized for 2 months. His early childhood was unremarkable with no history of recurrent nontuberculous infections, and all his childhood immunisations, including the bacille Calmette‐Guerin (BCG) vaccine, were up to date and uncomplicated. He reported no significant drug or toxin history. His parents were not related and there was no significant family history of recurrent or severe infections or hematological malignancy.

**FIGURE 2:**
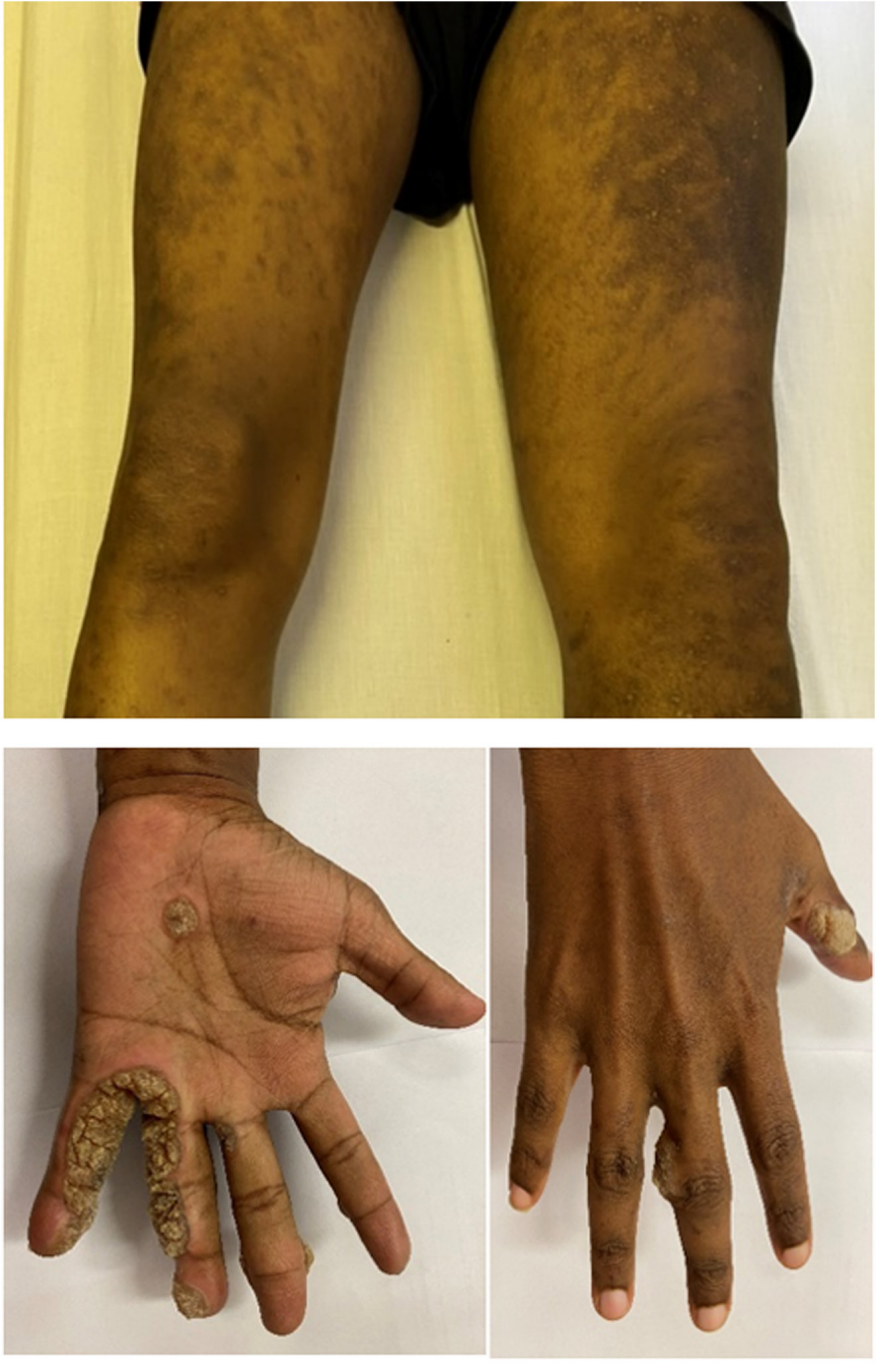
The top image shows lymphoedema of the left leg, and the bottom images show extensive verruca vulgaris of the fingers in a patient with GATA2 deficiency.

At presentation, the significant findings on clinical examination were extensive verrucae on both hands and feet, and chronic lymphoedema of the left leg (Figure 2). There were no skeletal abnormalities. His chest x‐ray was normal and in particular, showed no features of pulmonary alveolar proteinosis. He had severe pancytopenia with a low reticulocyte production index (RPI). There were no records of blood counts prior to this presentation. Blood counts, lymphocyte subsets and immunoglobulin fractions are shown in Table 1. Vaccine responses were normal.

**TABLE 1:** Blood cell counts, lymphocyte subsets and immunoglobulin levels of a patient at presentation to Clinical Hematology unit, Tygerberg Hospital and at diagnosis of GATA2 deficiency.

	At presentation^a^	At diagnosis		Reference range
Blood counts				
WCC	0.90 L	1.56 L		3.92**–**10.40 × 10^9^/L
Neutrophils	0.68 L	0.95 L		1.6**–**6.98 × 10^9^/L
Lymphocytes	0.20 L	0.53 L		1.40**–**4.20 × 10^9^/L
Monocytes	0.01 L	0.02 L		0.30**–**0.80 × 10^9^/L
Hemoglobin	4.2 L	13.2^b^ N		13.0**–**17.0 g/dL
MCV	93.5 N	105.0 H		83.1**–**101.6 fL
Platelets	46 L	76 L		171**–**388 × 10^9^/L
RPI	0.1 L			1**–**2
Vitamin B12	619 H			133–675 pmol/L
Serum folate	23.9 N			7.0–45.1 nmol/L
Lymphocyte subsets				
CD3		509 L	395 L	527**–**2846 cells/μL
CD4		46 L	136 L	332**–**1642 cells/μL
CD8		313 N	246 N	170**–**811 cells/μL
CD19		6 L	8 L	78**–**899 cells/μL
CD16/CD56		123 N	45 L	67**–**11,134 cells/μL
Immunoglobulin fractions				
IgG	14.01 N	11.99 N		7**–**16 g/L
IgA	1.44 N	1.00 N		0.7**–**4 g/L
IgM	0.58 N	0.98 N		0.4**–**2.3 g/L
Oxidative burst to PMA	29.0 L High fMLP suggesting infection. Repeat testing was required.	95.4 L		99**–**100%

Abbreviations: fMPL, formyl‐methionyl‐leucyl‐phenylalanine; H, high; Ig, immunoglobulin; L, low; MCV, mean cell volume; N, normal; PMA, phorbol 12‐myristate 13‐acetate; RPI, reticulocyte production index; WCC, white cell count.

^a^ At this time the patient had a disseminated non‐tuberculosis mycobacterium infection.

^b^ Transfusion support.

**FIGURE 3:**
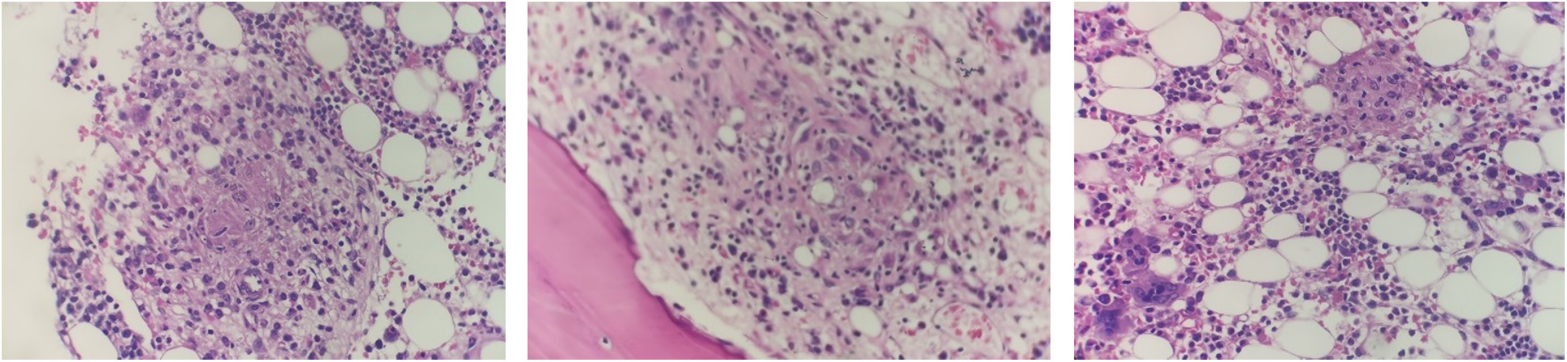
Bone marrow examination showing multiple small granulomas (original magnification 50×).

A BME showed a hypocellular bone marrow with trilineage dysplasia and multiple small granulomas (Figure 3). The karyotype was normal and fluorescence in situ hybridisation (FISH) was negative for deletion 5q31/monosomy 5 (MetaSystems Probes GmbH), deletion 7q22q31/monosomy 7 (MetaSystems Probes GmbH), deletion 20q12 (MetaSystems Probes GmbH), trisomy 8 (Vysis IGH/Myc/CEP 8 Tri‐Color, DF FISH Probe, Abbott) and loss of the Y chromosome (Vysis CEP X/Y DNA probe kit, Abbott). Mycobacterial culture of bone marrow aspirate showed acid‐fast bacilli, which was identified by polymerase chain reaction (PCR) as the NTM, *Mycobacterium intracellulare*.

The BME was repeated a year later as pancytopenia was not improving despite treatment for the NTM. The marrow was hypocellular with trilineage dysplasia and no granulomas, and karyotype remained normal. Given that the patient was on anti‐mycobacterial drugs that affect deoxyribonucleic acid (DNA) synthesis, there was caution to diagnose MDS in the absence of a clonal marker.

**FIGURE 4:**
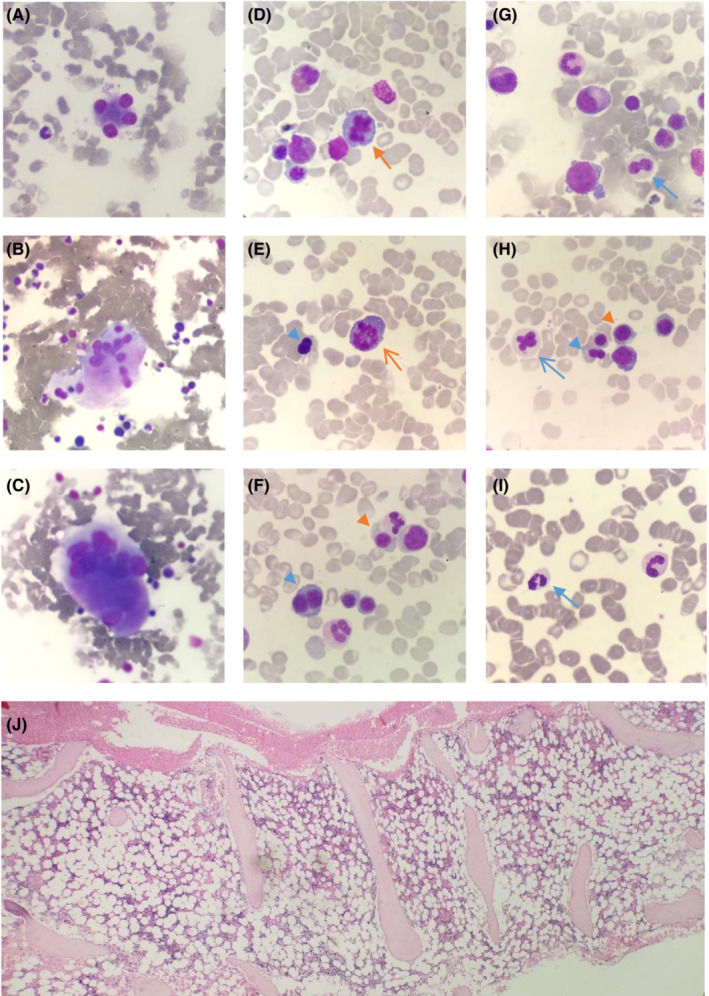
Bone marrow aspirate (A–I) and trephine (J) showing trilineage dysplasia and hypocellular marrow. Panels A‐C show multinucleate megakaryocytes, peripheralized lobes (original magnification 50×). Panels D–I show dysplasia in erythropoiesis and granulopoiesis (original magnification 100×). The orange arrowhead shows nuclear‐cytoplasmic asynchrony, the orange arrow shows karyorrhexis, the green arrow shows a mitotic figure, the blue arrowhead shows nuclear budding, and blue arrows show hypogranular neutrophils (open arrow) and hypogranular and hypolobated neutrophils (solid arrow). Panel J shows the hypocellular bone marrow trephine (original magnification 5×).

Once anti‐mycobacterial therapy was completed, the BME was repeated. Again, the marrow was hypocellular with persistent trilineage dysplasia (Figure 4), in keeping with a diagnosis of MDS with multilineage dysplasia (MDS‐MLD). Next‐generation sequencing (NGS) was performed on DNA and ribonucleic acid (RNA) extracted from the marrow sample using the Ion Torrent Oncomine™ Myeloid Research Assay and the Ion GeneStudio™ S5 System (ThermoFisher Scientific). It showed a loss of function *GATA2* variant with an allele frequency of 47.97% and two subclonal *STAG2* variants (Table 2). Given that the *GATA2* variant had an allelic frequency close to 50% and that the variant was described as a pathogenic on ClinVar (VCV000029719.5), a germline variant was considered most likely. The germline *GATA2* variant was confirmed by NGS on a skin biopsy, where the identical *GATA2* variant was detected (Table 2).

**TABLE 2:** Gene variants detected on bone marrow and skin analysis.

	Gene	Gene Variant and amino acid change	Effect on function	Allele frequency (%)	Cosmic ID
BMA	*GATA2* (NM_032638)	c.1009C>T p.(R337*)	Loss of function	47.97%	COSM6022403
	*STAG2* (NM_001042749)	c.832C>T p.(Q278*)	Loss of function	3.29%	COSM4167166
		c.2290C>T p.(Q764*)	Loss of function	12.77%	—
Skin	*GATA2* (NM_032638)	c.1009C>T p.(R337*)	Loss of function	48.85%	COSM6022403

*Note*: Variants were reported using Human Genome Variation Society (HGVS) notation. Variant calling was confirmed using the Catalogue of Somatic Mutations in Cancer (COSMICv96) and the National Center for Biotechnology Information: ClinVar databases.

Abbreviation: BMA, bone marrow aspirate.

Based on the above findings, the patient was diagnosed with GATA2 deficiency with an IEI and MDS‐MLD. The patient is currently being worked‐up for an allogeneic hematopoietic stem cell transplant (allo‐HSCT). His sister is a human leukocyte antigen (HLA) identical match and is negative for the *GATA2* variant. His parents also tested negative for the *GATA2* variant.

## 3 DISCUSSION

The important aspect of this case that alerted us to consider an IEI was the unusual presentation of the disseminated NTM infection in an immune‐competent individual. Additionally, MDS is uncommon in young adults. In this patient, both these unusual findings were explained by the diagnosis of germline GATA2 deficiency.

Germline *GATA2* variants that result in haplodeficiency of GATA2 have an autosomal dominant inheritance and may be familial (62%) or de novo (38%).^4^ Our patient has a de novo germline *GATA2* variant as his parents tested negative for the *GATA2* variant.

Originally, germline *GATA2* variants were identified as four separate clinical syndromes, however, due to the overlapping features with the subtypes, the clinical syndromes are now considered to be part of a single disorder known as GATA2 deficiency.^5^ Cohort studies of GATA2 deficiency highlighted that there is considerable variation in clinical phenotype, even within families that have the same variant, and that the mononuclear (monocyte, dendritic cell, B‐cell and NK‐cell) deficiency evolves over a period of years.^6^, ^7^ Cell counts in our patient showed a marked monocytopenia and profoundly reduced B‐cell count, in keeping with the laboratory findings of GATA2 deficiency.^6^, ^8^

In GATA2 deficiency, T‐cell populations are relatively well preserved.^8^ The relative preservation of T‐cell numbers, specifically cytotoxic T‐cells, is a feature that is seen in our patient. The maintenance of cytotoxic T‐cell counts represents the chronic antigen stimulation that drives the expression of CD8 memory cells.^4^, ^6^ While reduced NK‐cells are commonly described in GATA2 deficiency, our patient initially had a normal NK‐cell count.^4^ Preservation of NK‐cells may be due to NK‐cell memory in response to chronic antigen exposure, despite depletion of the naïve NK‐cell compartment and may explain why the NK‐cell counts were initially normal and were low on repeat testing.^9^

It is an evolving defective cell‐mediated immunity that results in the clinical manifestations and susceptibility to infection.^5^ Our patient presented with verucca vulgaris and NTM infection, which are the most common manifestations of the immunodeficiency described in GATA2 deficiency.^5^ Lymphoedema was also present, and frameshift or null mutations, as seen in our patient, has been shown to be associated with the development of lymphoedema.^6^ Furthermore, unlike other congenital forms of lymphoedema that are usually bilateral and present when children begin to walk, the lymphoedema associated with GATA2 deficiency is usually unilateral and can develop at a later age after a precipitating event, as is likely the case in our patient.^5^

MDS in early adulthood is uncommon; however, it occurs in 70% of persons with GATA2 deficiency.^10^ Transformation to AML or chronic myelomonocytic leukemia (CMML) occurs in 5.7%.^11^ MDS is typically preceded by immunodeficiency and marrow hypocellularity in adolescence, with cytopenias heralding the onset of MDS.^11^ The typical bone marrow pathological features include marrow hypocellularity, trilineage myeloid dysplasia and increased reticulin deposition.^12^ As seen in our patient, megakaryocytic disorder is most pronounced.^6^

Cytogenetic abnormalities are only seen in a quarter of adults with GATA2 deficiency and these include trisomy 8 and monosomy.^6^, ^7^, ^11^ However, these cytogenetic abnormalities are more common in patients with GATA2 deficiency that have MDS, seen in 68%.^11^ Our patient had a normal karyotype. This highlights the important role of molecular genetics to complement the morphological diagnosis of MDS.

Acquired somatic mutations are also only seen in a quarter of adults with GATA2 deficiency and these include variants in stromal antigen 2 (*STAG2*), additional sex combs like 1 (*ASXL1*) and DNA methyltransferase 3 alpha (*DNMT3A*).^11^ *ASXL1* mutations are more common in females while *STAG2* mutations are more common in males.^10^, ^11^ In accordance, our patient had *STAG2* mutations.

Allo‐HSCT remains the only curative option and reverses the phenotype of GATA2 deficiency through reconstitution of the immune system and hematopoietic compartment.^6^, ^13^ A critical step to the success of allo‐HSCT is the screening of family members for GATA2 variants, as has been performed for this patient.

This is the first case of GATA2 deficiency described in South Africa. The case study has highlighted the challenges in the diagnosis of MDS with a normal karyotype in patients with comorbidities or on drugs that are associated with non‐malignant dysplasia of bone marrow cells. The persistent pancytopenia that did not resolve despite optimal NTM management, was the alert in this case that further investigation for a hematological disorder was required. Furthermore, this case highlights the benefit of NGS not only to establish clonality of the malignancy but also to diagnose related predisposing conditions. Features of IEI and myeloid malignancy should prompt the investigation for germline variants.

## AUTHOR CONTRIBUTIONS

**Erica‐Mari Nell:** Conceptualization; data curation; methodology; writing – original draft. **Helena Cornellissen:** Data curation; methodology; writing – review and editing. **Katherine Hodkinson:** Conceptualization; data curation; methodology; writing – original draft. **Michael F. Urban:** Data curation; writing – review and editing. **Fatima Cassim Bassa:** Methodology; supervision. **Fatima Bibi Fazel:** Data curation; methodology; writing – original draft. **Tracey Wiggill:** Data curation; methodology; writing – review and editing. **Semira Irusen:** Data curation. **Zivanai C. Chapanduka:** Supervision; writing – review and editing.

## FUNDING INFORMATION

No funding was received.

## CONFLICT OF INTEREST STATEMENT

The authors have no conflicts of interest to disclose.

## CONSENT

The patient signed informed consent for his case to be presented and appear in a journal article.

## ETHICS APPROVAL STATEMENT

Ethics approval was obtained from Stellenbosch University Health Research Ethics Committee (C22/07/018).

## Data Availability

All data are available in the manuscript.
